# The Effects of CSF Neurogranin and *APOE* ε4 on Cognition and Neuropathology in Mild Cognitive Impairment and Alzheimer’s Disease

**DOI:** 10.3389/fnagi.2021.667899

**Published:** 2021-04-27

**Authors:** Yulan Fan, Ying Gao, Joseph Therriault, Jing Luo, Maowen Ba, Hua Zhang, the Alzheimer’s Disease Neuroimaging Initiative

**Affiliations:** AbbVie, Alzheimer’s Association, Alzheimer’s Drug Discovery Foundation, Araclon Biotech, BioClinica, Inc., Biogen, Bristol-Myers Squibb Company, CereSpir, Inc., Cogstate; Eisai Inc., Elan Pharmaceuticals, Inc., Eli Lilly and Company, EuroImmun, F. Hoffmann-La Roche Ltd., and its affiliated company Genentech, Inc., Fujirebio, GE Healthcare, IXICO Ltd., Janssen Alzheimer Immunotherapy Research and Development, LLC., Johnson & Johnson Pharmaceutical Research and Development, LLC., Lumosity, Lundbeck; Merck & Co., Inc., Meso Scale Diagnostics, LLC., NeuroRx Research, Neurotrack Technologies, Novartis Pharmaceuticals Corporation, Pfizer Inc., Piramal Imaging, Servier, Takeda Pharmaceutical Company, and Transition Therapeutics; ^1^Department of Neurology, The First Affiliated Hospital of Chongqing Medical University, Chongqing, China; ^2^General Medical Wards, The Affiliated Children’s Hospital of Chongqing Medical University, Chongqing, China; ^3^The McGill University Research Centre for Studies in Aging, McGill University, Montreal, QC, Canada; ^4^Department of Neurology, Yantai Yuhuangding Hospital Affiliated to Qingdao Medical University, Yantai, China

**Keywords:** Alzheimer’s disease, apolipoprotein E ε4, mild cognitive impairment, neurogranin, cerebrospinal fluid

## Abstract

Cerebrospinal fluid (CSF) measurements of neurogranin (Ng) have emerged as a promising biomarker for cognitive decline in mild cognitive impairment (MCI) and Alzheimer’s disease (AD). The apolipoprotein E ε4 (*APOE* ε4) allele is by far the most consistent genetic risk factor for AD. However, it is not known whether the pathophysiological roles of Ng in MCI or AD are related to *APOE*ε4. We stratified 250 participants from the Alzheimer’s Disease Neuroimaging Initiative (ADNI) database into cognitively normal (CN) ε4 negative (CN ε4−), CN ε4 positive (CN ε4+), MCI ε4 negative (MCI ε4−), MCI ε4 positive (MCI ε4+), AD ε4 negative (AD ε4−), and AD ε4 positive (AD ε4+). CSF Ng levels were significantly increased in *APOE* ε4 carriers compared to *APOE* ε4 non-carriers with MCI. In addition, CSF Ng identified MCI ε4+ versus CN ε4−, but not MCI ε4− versus CN ε4−. Similarly, CSF Ng negatively correlated with Mini-Mental State Examination (MMSE) scores at baseline in the MCI ε4+ group. Our findings support the use of CSF Ng as a biomarker of synaptic pathology for AD. We propose that the roles of CSF Ng in the pathophysiology of MCI may be related to *APOE* ε4.

## Introduction

Alzheimer’s disease (AD) is a leading cause of dementia. Extracellular depositions of amyloid beta (Aβ) peptides and intracellular neurofibrillary tangles composed of hyperphosphorylated tau are the major pathological characteristics of AD (Jack et al., 2018). The etiology and pathogenesis of AD are still unclear, but more than 15 genome-wide studies have indicated that apolipoprotein E ε4 (*APOE* ε4) allele is related to AD and is by far the most consistent genetic risk factor (Bertram et al., 2010; Lambert et al., 2013). Compared with non-carriers, *APOE* ε4 carriers tend to show an accelerated cognitive decline.

Synaptic degeneration and dysfunction are critical pathological events in AD (DeKosky and Scheff, 1990). Synaptic loss has been identified as an important contributor to progressive cognitive decline and an early feature in AD progression (DeKosky and Scheff, 1990). Furthermore, compared with Aβ deposits and neurofibrillary tangles, synapse loss in the hippocampus is more closely related to the degree of cognitive impairment (Scheff et al., 2006, 2007). Therefore, synaptic protein biomarkers are promising tools for the early diagnosis of AD. In addition, in clinical trials of disease-modifying therapies for AD, synaptic biomarkers can potentially monitor disease progression and evaluate effects of drugs on synaptic dysfunction and degeneration. Neurogranin (Ng), a post synaptic protein with 78-amino acids, plays a critical role in long-term potentiation and memory consolidation (Gerendasy and Sutcliffe, 1997; Pak et al., 2000; Huang et al., 2004), where it regulates the concentrations of calmodulin in response to intracellular calcium concentrations following neuronal excitation (Baudier et al., 1991; Xia and Storm, 2005; Díez-Guerra, 2010). Several studies have suggested that the concentrations of Ng are increased in cerebrospinal fluid (CSF) (De Vos et al., 2015; Hellwig et al., 2015; Kvartsberg et al., 2015b; Portelius et al., 2015; Janelidze et al., 2016) and decreased in the brain of patients with AD (Davidsson and Blennow, 1998; Reddy et al., 2005; Kvartsberg et al., 2019). In mild cognitive impairment (MCI) patients, high CSF Ng concentrations at baseline can predict cognitive decline during clinical follow-up (Portelius et al., 2015). Furthermore, high CSF Ng levels at baseline are associated with longitudinal reductions in hippocampal volume and cortical glucose metabolism during clinical follow-up in MCI (Portelius et al., 2015). In addition, in MCI patients who progress to dementia, increased CSF Ng concentrations are related to accelerated deterioration of Alzheimer’s disease assessment scale (ADAS) (Portelius et al., 2015). Another study has also demonstrated CSF Ng is correlated with brain atrophy (Tarawneh et al., 2016).

However, the relationship between *APOE*ε4 and Ng is poorly understood, and it is not known whether the above-mentioned roles of Ng are related to *APOE*ε4. In this study, we show the results of CSF Ng in the Alzheimer’s Disease Neuroimaging Initiative (ADNI) cohort, including the cognitively normal (CN) control, MCI participants, and AD-induced dementia. We verified the hypotheses that CSF Ng is increased in *APOE* ε4 positive individuals compared with *APOE* ε4 negative participants in each diagnostic group. We also report that CSF Ng reflects neurodegeneration dependently of *APOE* ε4.

## Materials and Methods

### Database Description

Data used in preparation of this article were obtained from the ADNI database. The ADNI was launched in 2003 as a public-private partnership, led by Principal Investigator Michael W. Weiner, MD. The primary goal of ADNI has been to test whether serial MRI, positron emission tomography (PET), biological markers, and clinical and neuropsychological assessments can be combined to measure the progression of MCI and early AD. Further information can be found at http://www.adni-info.org (Zhang et al., 2018).

From the database, we selected all subjects between 55 and 90 (inclusive) years of age who had completed a lumbar puncture, clinical and neuropsychological evaluations, MRI, and FDG-PET. According to clinical and behavioral measures provided by ADNI, the individuals included in the study were classified as CN (*n* = 65), MCI (*n* = 122), and AD (*n* = 63). Individuals who have at least one ε4 allele were considered as ε4 carriers. According to whether the subjects carried ε4, they were divided into CN ε4 negative (CN ε4−, *n* = 48), CN ε4 positive (CN ε4+, *n* = 17), MCI ε4 negative (MCI ε4−, *n* = 50), MCI ε4 positive (MCI ε4+, *n* = 72), AD ε4 negative (AD ε4−, *n* = 17), and AD ε4 positive (AD ε4+, *n* = 46).

### Classification Criteria

Cognitively normal individuals had MMSE scores ranging between 24 and 30, and a Clinical Dementia Rating scale (CDR) score of 0 (Folstein et al., 1975; Berg, 1988). Subjects with MCI had subjective memory complaints, an MMSE score from 24 to 30, a CDR of 0.5, remained activities of daily living, and no dementia (Aisen et al., 2010). Apart from the NINCDS/ADRDA criteria, the MMSE scores of AD dementia patients were between 20 and 26, and the CDR was 0.5 or 1.0 (Tierney et al., 1988; Zhang et al., 2018) [For more information on inclusion/exclusion criteria, please visit www.adni-info.org (accessed December 2020)].

### Standard Protocol Approvals and Patient Consents

The ADNI study was approved by the Institutional Review Boards of all the participating institutions. Informed written consent was obtained from all subjects at each center (Zhang et al., 2018).

### Analyses of CSF Aβ42, T-Tau, P-Tau, and Ng

As described elsewhere, the use of multiple xMAP-Luminex platforms and Innogenetics INNO-BIA AlzBio3 immunoassay reagents were used for the quantification of CSF Aβ42, T-tau, and P-tau phosphorylated at threonine 181 (Shaw et al., 2009). The Ng-specific monoclonal antibody Ng7 was used as the coating antibody, and the polyclonal Ng anti-rabbit antibody was used as the detection antibody. Electrochemiluminescence technology was used to analyze CSF-Ng. Values are expressed in pg/ml. All CSF data used in the present study were from the ADNI files “UPENNBIOMK5-8.csv” and “BLENNOWCSFNG.csv,” (accessed December 2020). For more detailed information on the ADNI method for CSF collection, quantification, and quality control procedures, please visit www.adni-info.org.

### Cognitive Evaluation

Mini-Mental State Examination and ADAS-Cog 11 scores were used to assess overall cognitive abilities. Because of the lack availability of some follow-up data, we only collected baseline scores of MMSE and ADAS-cog 11. The data utilized in the present study were from ADNI files “MMSE.csv” and “ADAS_ADNI1.csv,” (accessed December 2020).

### Neuroimaging Methods

Hippocampal and ventricular volumes were used to assess neurodegeneration. These data came from the ADNI files “FOXLABBSI_08_04_17.csv” and “UCSDVOL.csv,” (accessed December 2020). We also only selected baseline imaging data because there were too many missing data during the follow-up period. ADNI’s neuroimaging methods have been described in detail elsewhere (Risacher and Saykin, 2013). For more detailed information about ADNI image acquisition and processing, please visit www.adni-info.org/methods.

### FDG-PET

Acquisition and processing of PET imaging data in ADNI is described in detail elsewhere^1^ ^,2^, respectively. For detailed instructions, see Landau et al. (2012). Briefly, FDG standardized uptake value ratio (SUVR) value for each participant was estimated as the mean SUVR of the lateral and medial prefrontal, anterior cingulate, posterior cingulate, lateral parietal, and lateral temporal cortices.

### Statistical Methods

On baseline demographics, chi-square analyses, and analysis of covariance (ANOVA) were employed for categorical variables and for continuous variables, respectively. Multivariable linear regression (adjusted for age and gender) were used to detect CSF Ng levels in each diagnostic group. To assess the potential influence of *APOE* ε4, we used an interaction term between APOE ε4 positivity and diagnosis as a predictor in the statistical models.

Spearman correlations were used to assess relationships between Ng and other core AD biomarkers. For each biomarker, ROC analyses (adjusted for age and gender) were employed for diagnostic accuracy (area under the receiver operator characteristics curve, AUC). Bootstrapping method was used to assess the potential differences between two AUCs derived from all pairs of two different biomarkers.

The relationships between Ng and the incidence of AD were evaluated by calculating hazard ratios (HR) with 95% CIs using Cox proportional hazard regression analyses adjusting for age, gender, and Aβ42. In Cox proportional hazards regression analysis, Ng status was divided into two groups according to the median of each biomarker.

For MMSE, ADAS-cog 11, hippocampal volume, ventricular volume, and FDG-PET SUVR, linear mixed effects models were used to obtain intercept (baseline values). The intercept was then used as outcomes in subsequent linear regression models with Ng as predictor (adjusted for age, gender, and Aβ42; and for education for MMSE and ADAS-cog 11; and for intracranial volumes for hippocampal volumes and ventricular volumes) within diagnostic groups. All outcome variables in linear mixed-effects models used normalized values to facilitate comparisons between modalities (Zhang et al., 2018). All statistics were done using R (v. 3.4.2) and SPSS version 21. The statistical significance of all analyses was defined as *p* < 0.05.

## Results

### Characteristics of Subjects

Demographics, clinical, and biomarker features of the study subjects are shown in Table 1. We did not observe statistically significant differences in age or education among the groups. Compared with the CN ε4− and AD ε4− groups, there were significantly fewer female subjects in the MCI ε4− group (*p* = 0.007, *p* = 0.024, respectively). Between CN ε4− and CN ε4+ (*p* = 0.001) and between MCI ε4− and MCI ε4+ (*p* < 0.001), CSF Aβ42 levels were significantly lower in *APOE* ε4 positive subjects. There was no similar phenomenon between AD ε4− and AD ε4+ (*p* = 0.438). Between MCI ε4− and MCI ε4+, CSF total-tau (T-tau) (*p* = 0.002) and phosphorylated-tau (P-tau) (*p* = 0.001) in *APOE* ε4 positive participants increased significantly. However, there was no similar finding between CN ε4− and CN ε4+, or between AD ε4− and AD ε4+. MMSE scores were lower in MCI ε4−, MCI ε4+, AD ε4−, and AD ε4+ groups compared with CN ε4− and CN ε4+ subjects, and lower in AD ε4− and AD ε4+ groups compared with MCI ε4− and MCI ε4+. ADAS-Cog 11 scores were higher in MCI ε4−, MCI ε4+, AD ε4−, and AD ε4+ compared to CN ε4− and CN ε4+, and higher in AD ε4− and AD ε4+ compared with MCI ε4− and MCI ε4+.

**TABLE 1:** Main demographics of subjects at baseline.

**Characteristics**	**CN ε4− (*n* = 48)**	**CN ε4+ (*n* = 17)**	**MCI ε4− (*n* = 50)**	**MCI ε4+ (*n* = 72)**	**AD ε4− (*n* = 17)**	**AD ε4+ (*n* = 46)**
Age (years)	74.9 (0.7)	75.7 (1.4)	72.9 (1.2)	73.0 (0.8)	73.1 (2.3)	74.2 (1.1)
Sex (female %)	26(54.2%)^c^	6(35.3%)	14(28.0%)^a,e^	31(43.1%)	10(58.8%)^c^	21(45.7%)
Education (years)	15.7 (0.4)	15.7 (0.9)	15.5 (0.5)	15.7 (0.3)	15.7 (0.7)	14.7 (0.4)
CSF Aβ42 (pg/ml)	1128.4(53.8)^b,c,d,e,f^	768.2(70.0)^a,f^	902.2(57.2)^a,d,f^	632.5(30.3)^a,c^	729.5(67.7)^a,f^	538.7(20.7)^a,b,c,e^
CSF T-tau (pg/ml)	215.6(8.0)^d,e,f^	267.8(25.5)^d,e,f^	273.1(16.8)^d,e^	351.9(14.3)^a,b,c^	391.2(42.1)^a,b,c^	344.1(15.3)^a,b^
CSF P-tau (pg/ml)	19.9(0.9)^d,e,f^	26.4(2.8)^d,e^	26.4(1.9)^d,e^	36.1(1.7)^a,b,c^	39.4(4.8)^a,b,c^	34.7(1.6)^a^
MMSE	29.3(0.1)^c,d,e,f^	29.0(0.2)^c,d,e,f^	26.8(0.3)^a,b,e,f^	26.9(0.2)^a,b,e,f^	23.9(0.4)^a,b,c,d^	23.1(0.3)^a,b,c,d^
ADAS-cog 11	6.4(0.4)^c,d,e,f^	7.5(0.8)^c,d,e,f^	10.9(0.7)^a,b,e,f^	12.4(0.6)^a,b,e,f^	18.8(1.7)^a,b,c,d^	18.4(0.8)^a,b,c,d^

*Measurement data are expressed by mean and standard error. *P-*values indicate the values assessed with analyses of variance for each variable except gender, where a contingency chi-square was performed. *Post hoc* analysis provided significant differences between groups: ^*a*^from CN ε4−; ^*b*^from CN ε4+; ^*c*^from MCI ε4−; ^*d*^from MCI ε4+; ^e^from AD ε4−; ^*f*^from AD ε4+ (at threonine 181). CSF, cerebrospinal fluid; T-tau, total-tau; P-tau, phosphorylated-tau at threonine 181; MMSE, Mini–Mental State Examination; ADAS-cog 11, Alzheimer’s disease assessment scale-cog 11; CN, cognitively normal; MCI, mild cognitive impairment; AD, Alzheimer’s disease.*

### CSF Ng Concentrations in *APOE* ε4 Positive and Negative Participants in Every Diagnostic Group

**FIGURE 1:**
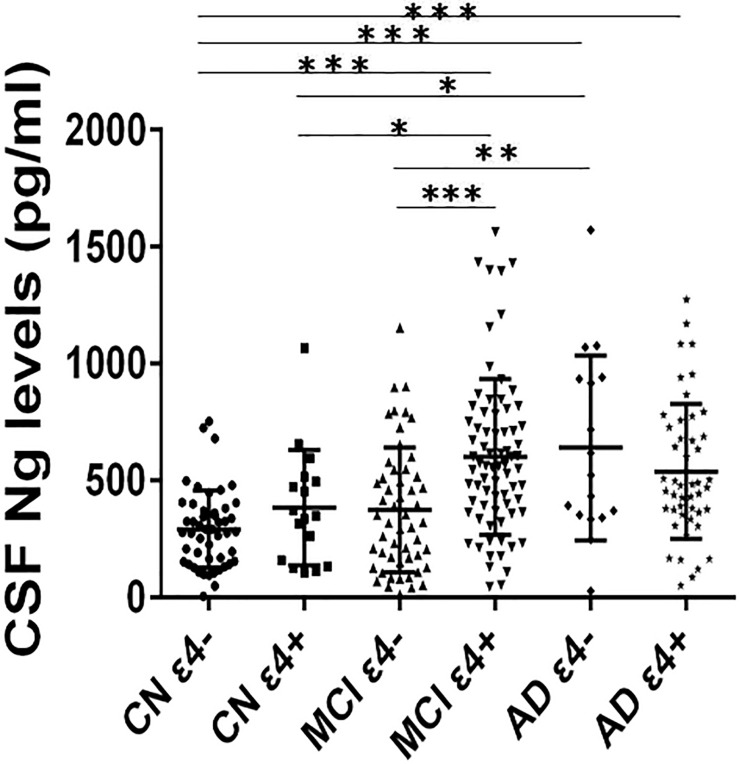
CSF Ng levels in different diagnostic groups. The subjects included in the study were classified as CN ε4–, CN ε4+, MCI ε4–, MCI ε4+, AD ε4–, and AD ε4+. Differences between groups were tested by multiple-variable linear regression, adjusted for age and sex. ^∗^*P* < 0.05; ^∗∗^*P* < 0.01; ^∗∗∗^*P* < 0.0001. Ng, neurogranin; CN, healthy controls; MCI, mild cognitive impairment; AD, Alzheimer’s disease.

Cerebrospinal fluid Ng levels were significantly higher in MCI ε4+, AD ε4−, and AD ε4+ (all *p* < 0.001) compared to CN ε4− (Figure 1). Higher Ng concentrations were observed in MCI ε4+ (*p* < 0.05) and AD ε4− (*p* < 0.05) compared to CN ε4+ (Figure 1). Between MCI ε4− and MCI ε4+, CSF Ng levels in *APOE* ε4 positive participants increased significantly (*p* < 0.001) (Figure 1), but there were no statistically significant differences between CN ε4− and CN ε4+ as well as between AD ε4− and AD ε4+ (Figure 1).

### CSF Ng Levels in Relation to CSF Aβ and Tau

**FIGURE 2:**
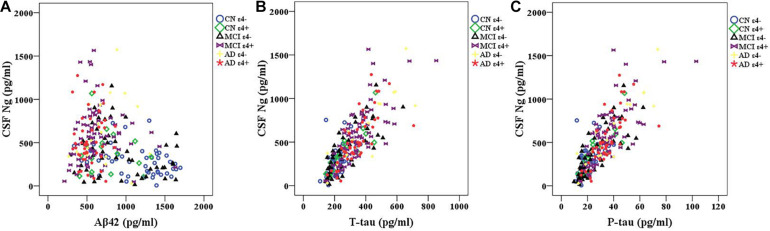
CSF Ng in relation to Aβ42 and tau biomarkers. Spearman correlations were used to assess relationships between Ng and Aβ42 and tau biomarkers. Correlations between CSF Ng and Aβ42 **(A)** and tau biomarkers **(B,C)** in different diagnostic groups. Ng, neurogranin; CN, healthy controls; MCI, mild cognitive impairment; AD, Alzheimer’s disease.

We observed negative correlations between Ng and Aβ42 in CN ε4− participants (*r* = −0.353, *p* = 0.014) (Figure 2A). However, there were no statistically significant relationships between Ng and Aβ42 in CN ε4+ (*r* = −0.095, *p* = 0.717), MCI ε4− (*r* = −0.194, *p* = 0.177), MCI ε4+ (*r* = −0.023, *p* = 0.845), AD ε4− (*r* = 0.172, *p* = 0.509), and AD ε4+ (*r* = 0.080, *p* = 0.596) (Figure 2A). Ng was strongly correlated with T-tau and P-tau in CN ε4− (*r* = 0.550, *p* < 0.001; *r* = 0.519, *p* < 0.001, respectively), CN ε4+ (*r* = 0.858, *p* < 0.001; *r* = 0.841, *p* < 0.001, respectively), MCI ε4− (*r* = 0.799, *p* < 0.001; *r* = 0.784, *p* < 0.001, respectively), MCI ε4+ (*r* = 0.746, *p* < 0.001; *r* = 0.726, *p* < 0.001, respectively), AD ε4− (*r* = 0.869, *p* < 0.001; *r* = 0.906, *p* < 0.001, respectively), and AD ε4+ subjects (*r* = 0.747, *p* < 0.001; *r* = 0.726, *p* < 0.001, respectively) (Figures 2B,C).

### Diagnostic Accuracy of CSF Ng, T-Tau, and P-Tau

**FIGURE 3:**
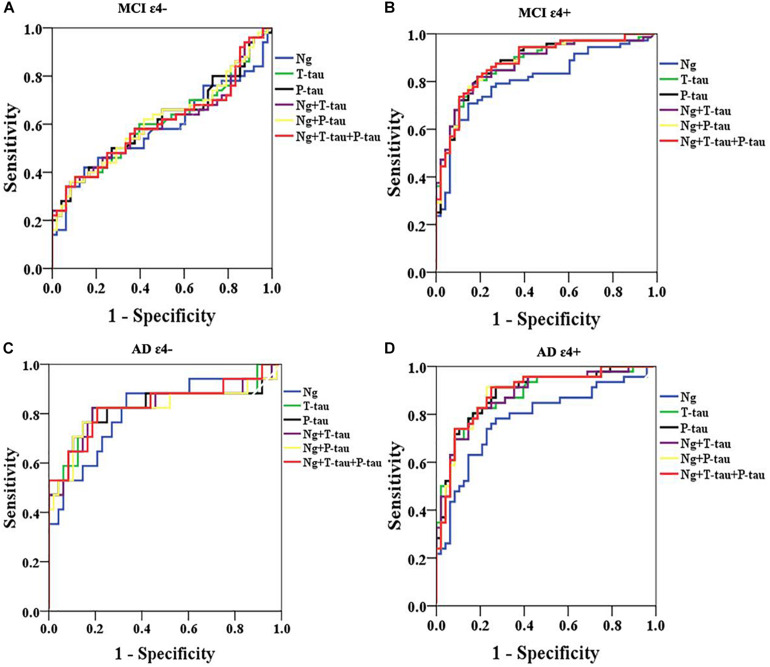
ROC analyses. ROC analyses were performed to test the CSF biomarkers in relation to clinical diagnoses for MCI ε4– **(A)**, MCI ε4+ **(B)**, AD ε4– **(C)**, and AD ε4+ **(D)**. ROC analyses were adjusted for age and gender. Ng, neurogranin.

Receiver operating curve (ROC) analyses were carried out to detect CSF biomarkers related to clinical diagnoses in CN ε4+, MCI ε4−, MCI ε4+, AD ε4−, and AD ε4+. Compared to CN ε4−, CSF Ng, T-tau, and P-tau had high diagnostic accuracy for MCI ε4+ (Table 2 and Figure 3B), AD ε4− (Table 2 and Figure 3C), and AD ε4+ (Table 2 and Figure 3D) but not MCI ε4− (Table 2 and Figure 3A). CSF Ng did not have diagnostic accuracy for CN ε4+ (Table 2). The diagnostic accuracy of CSF Ng for MCI ε4+, AD ε4−, and AD ε4+ was almost the same as that of CSF T-tau and P-tau (Table 2 and Figures 3B–D). However, compared to T-tau and P-tau, combination of Ng, T-tau or P-tau did not significantly improve the diagnostic accuracy for MCI ε4−, MCI ε4+, AD ε4−, and AD ε4+ (Table 2 and Figures 3A–D).

**TABLE 2:** AUC of CSF biomarkers.

	**Ng**	**T-tau**	**P-tau**	**Ng+ T-tau**	**Ng+ P-tau**	**Ng+ T-tau+ P-tau**
CN ε4+	0.613 (0.443–0.782) (*p* = 0.170)	0.624 (0.450–0.799) (*p* = 0.130)	0.672 (0.516–0.827) (*p* = 0.037)	0.626 (0.453–0.800) (*p* = 0.124)	0.669 (0.513–0.825) (*p* = 0.039)	0.718 (0.578–0.859) (*p* = 0.008)
MCI ε4–	0.585 (0.469–0.700) (*p* = 0.148)	0.606 (0.493–0.720) (*p* = 0.070)	0.613 (0.498–0.724) (*p* = 0.058)	0.600 (0.486–0.715) (*p* = 0.087)	0.612 (0.500–0.725) (*p* = 0.055)	0.603 (0.489–0.717) (*p* = 0.078)
MCI ε4+	0.808 (0.730–0.887) (*p* < 0.001)	0.875 (0.812–0.937) (*p* < 0.001)	0.881 (0.819–0.943) (*p* < 0.001)	0.876 (0.814–0.938) (*p* < 0.001)	0.881 (0.820–0.942) (*p* < 0.001)	0.881 (0.820–0.942) (*p* < 0.001)
AD ε4–	0.809 (0.676–0.941) (*p* < 0.001)	0.827 (0.686–0.968) (*p* < 0.001)	0.824 (0.677–0.970) (*p* < 0.001)	0.825 (0.683–0.967) (*p* < 0.001)	0.819 (0.672–0.965) (*p* < 0.001)	0.833 (0.699–0.968) (*p* < 0.001)
AD ε4+	0.783 (0.687–0.878) (*p* < 0.001)	0.879 (0.809–0.949) (*p* < 0.001)	0.888 (0.821–0.955) (*p* < 0.001)	0.880 (0.811–0.949) (*p* < 0.001)	0.889 (0.822–0.956) (*p* < 0.001)	0.888 (0.821–0.956) (*p* < 0.001)

*AUC, area under the receiver operator characteristics curve; Ng, neurogranin; T-tau, total-tau; P-tau, phosphorylated-tau at threonine 181; CN, cognitively normal; MCI, mild cognitive impairment; AD, Alzheimer’s disease.*

### CSF Ng and Conversion From CN to MCI or AD and From MCI to AD

**FIGURE 4:**
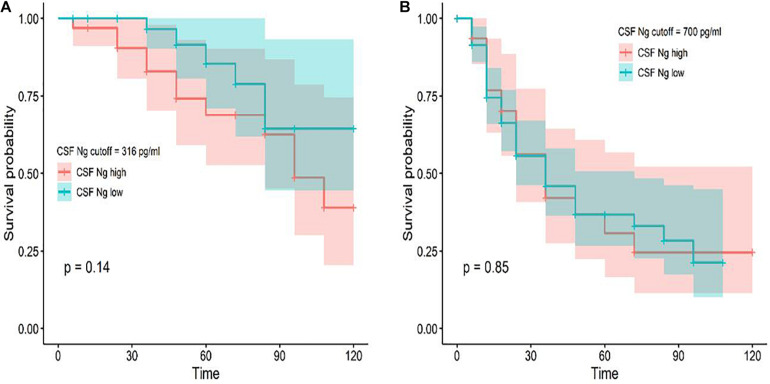
Baseline CSF measures of Ng as predictors of conversion from CN to MCI or AD and MCI to AD. Cox proportional hazard regression analyses were used to evaluate the relationships between Ng and the incidence of AD. Conversion from CN to MCI or AD **(A)** and MCI to AD **(B)** as a function of CSF Ng measures (dichotomized at the median values) are shown. Analyses were adjusted for age, education, and gender. Cutoff values were 316 pg/ml (CN) and 700 pg/ml (MCI) for Ng; Ng, neurogranin.

Among the subjects with longitudinal assessments, 18 CN individuals progressed to MCI or AD and 73 MCI participants progressed to AD during follow-up. We investigated whether CSF Ng predicted conversion from CN to MCI or AD and from MCI to AD. Cox proportional hazard models were performed for Ng as a continuous variable. HRs were then calculated for Ng as a dichotomized variable using median values of Ng as a threshold (adjusting for education, gender, and age). CSF Ng did not significantly predict conversion from CN to MCI or AD (Figure 4A), or from MCI to AD (Figure 4B).

### CSF Ng and *APOE* ε4 in Relation to Cognition

**FIGURE 5:**
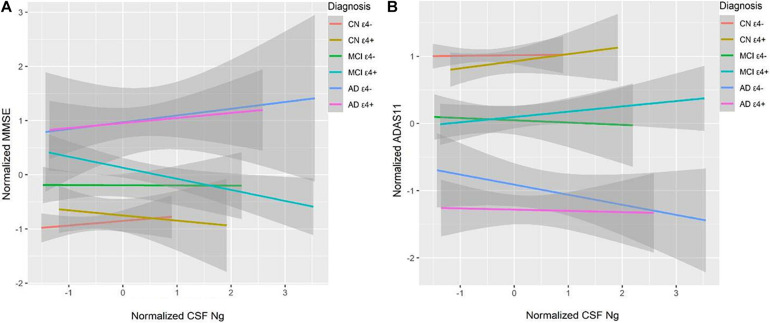
CSF Ng in relation to cognition. MMSE and ADAS-Cog 11 scores were used to assess overall cognitive abilities. MMSE **(A)** and ADAS-Cog 11 **(B)** at baseline as a function of baseline CSF Ng in different diagnostic groups. CSF Ng levels are normalized. CSF, cerebrospinal fluid; Ng, neurogranin; CN, cognitively normal; MCI, mild cognitive impairment; AD, Alzheimer’s disease.

High CSF Ng levels were related to lower Mini–Mental State Examination (MMSE) scores at baseline in the MCI ε4+ group (β = −0.18, *p* = 0.036), but not in the MCI ε4− group, or other groups (Figure 5A). We did not observe significant correlations between CSF Ng and ADAS-cog 11 at baseline in any diagnostic group (Figure 5B). Although there was a trend for associations between CSF Ng and with ADAS-cog 11 in the AD ε4− group, this did not reach statistical difference (β = −0.22, *p* = 0.064) (Figure 5B).

### CSF Ng and *APOE* ε4 in Relation to Brain Structure

**FIGURE 6:**
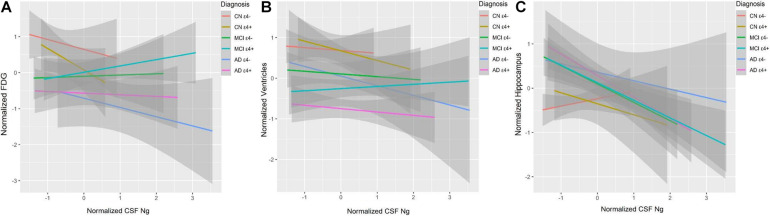
CSF Ng in relation to brain structure and metabolism. FDG was used to evaluate metabolism. Hippocampal and ventricular volumes were used to assess neurodegeneration. FDG **(A)**, ventricular volumes **(B)**, and hippocampal volumes **(C)** at baseline as a function of baseline CSF Ng in different diagnostic groups. CSF Ng levels are normalized. CSF, cerebrospinal fluid; Ng, neurogranin; FDG, 18F-Fluorodeoxyglucose; CN, cognitively normal; MCI, mild cognitive impairment; AD, Alzheimer’s disease.

Cerebrospinal fluid Ng did not correlate with baseline 18F-fluorodeoxyglucose-positron emission tomography (FDG-PET) or ventricular volumes in different diagnostic groups (Figures 6A,B). High CSF Ng levels were related to low hippocampal volumes in the MCI ε4− (β = −0.39, *p* = 0.007), the MCI ε4+ (β = −0.25, *p* = 0.036), and the AD ε4+ (β = −0.42, *p* = 0.003) (Figure 6C), but in the AD ε4− and other groups, no such associations were found (Figure 6C).

## Discussion

This study assessed the characteristics of CSF Ng in individuals with MCI or AD from the ADNI database. The study has four main findings: first, the levels of Ng in *APOE* ε4 positive participants were significantly higher in MCI ε4+ compared with MCI ε4−. However, there was no similar finding between CN ε4− and CN ε4+, or between AD ε4− and AD ε4+. Secondly, CSF Ng was strongly related to T-tau and P-tau but not Aβ42 in every diagnostic group. Our third main finding is that CSF Ng had almost identical diagnostic accuracy for MCI ε4+, AD ε4−, and AD ε4+ as did with T-tau and P-tau. However, the combination of Ng, T-tau or P-tau did not significantly improve diagnostic accuracy. Finally, high CSF Ng levels only associated with low MMSE scores at baseline in the MCI ε4+ group. Moreover, CSF Ng correlated with hippocampal volumes at baseline in the MCI ε4−, MCI ε4+, and AD ε4+ groups.

Previous studies have shown that, compared with healthy controls, CSF Ng was significantly elevated in MCI and AD, and MCI participants who progressed to dementia had higher concentrations of CSF Ng than stable MCI subjects (Kvartsberg et al., 2015a; Portelius et al., 2015). To assess whether CSF Ng levels can be used to identify MCI and AD subjects with potential *APOE* ε4 carriership, each group was divided into *APOE* ε4−positive and *APOE* ε4−negative in the present study. The concentrations of CSF Ng were significantly increased in the MCI ε4+, AD ε4−, and AD ε4+ groups compared to CN ε4− group. Interestingly, higher CSF Ng concentrations were observed in the MCI ε4+ group than MCI ε4− group, but there was no similar finding between CN ε4− and CN ε4+ and between AD ε4− and AD ε4+, suggesting that CSF Ng may be an early biomarker of AD-related synaptic degeneration (Portelius et al., 2015), and suggesting the roles of CSF Ng in the pathophysiology of MCI may be related to *APOE* ε4 status. However, CSF Aβ42 concentrations were significantly different between ε4− and ε4+ in every group. Therefore, it was necessary to further explore whether the roles of CSF Ng were being modulated in part by Aβ rather than *APOE* ε4 alone.

Previous studies demonstrated that CSF Ng is particularly elevated in individuals with MCI and AD with abnormal Aβ (Portelius et al., 2015; Wang, 2019). However, the relationship between CSF Ng and Aβ pathology remains controversial. Some studies have shown that CSF Ng positively or negatively correlated with Aβ42 or Aβ40 in AD patients (De Vos et al., 2015; Hellwig et al., 2015; Janelidze et al., 2016; Mattsson et al., 2016). Moreover, other researchers have reported that the CSF Ng levels did not correlate to Aβ42 in AD samples (Hellwig et al., 2015; Sanfilippo et al., 2016). In the present study, except for CN ε4− group, no relationship between CSF Ng and Aβ42 was observed in other diagnostic groups. Most likely, it indicates that the synaptic degeneration is weakly associated with the axonal damage induced by Aβ. In clinical trials, the failure of anti-amyloid therapy to reduce or reverse the decline of cognitive ability has aroused doubts about the Aβ cascade theory. Interestingly, some studies have demonstrated the role of Aβ in neuroprotection, synaptic function and memory consolidation (Lazarevic et al., 2017; Finnie and Nader, 2020). These beneficial effects are Aβ level and species specificity (Karisetty et al., 2020). Picomolar levels and monomers proved to be beneficial, while high levels and soluble oligomers proved to be harmful (Karisetty et al., 2020). These findings emphasize the need to understand the physiological and pathological roles of Aβ in order to improve current amyloid based therapeutic strategies (Karisetty et al., 2020). As AD is a multifactorial disease, targeting AD related processes such as tau pathology, synaptic activity, neural epigenetic regulation of AD associated genes, and inflammatory responses may provide alternative treatment strategies (Karisetty et al., 2020). In line with previous studies (De Vos et al., 2015; Hellwig et al., 2015; Portelius et al., 2015; Janelidze et al., 2016; Mattsson et al., 2016; Sanfilippo et al., 2016; Wang, 2019; Xue et al., 2020), we have found that elevated levels of CSF Ng were correlated with T-tau and P-tau in every diagnostic group. Interestingly, T-tau and P-tau concentrations were notably increased in *APOE* ε4 carriers compared to *APOE* ε4 non-carriers in MCI, but there was no significant difference in the concentrations of T-tau and P-tau between *APOE* ε4 carriers and *APOE* ε4 non-carriers in CN and AD. These findings suggest that synaptic degeneration especially in MCI subjects may be related to the axonal damage induced by tau or *APOE* ε4. It is important to consider that relationships between CSF biomarkers (such as Ng, Aβ, T-tau, and P-tau) within MCI populations cannot exclude the possibility that different pathogenic processes are involved.

We next sought to test whether CSF Ng could improve the differential diagnosis of MCI and AD dementia in comparison to the traditional AD biomarkers, such as CSF T-tau and P-tau. All biomarkers identified MCI ε4+ versus CN ε4−, AD ε4− versus CN ε4−, and AD ε4+ versus CN ε4−, but not MCI ε4− versus CN ε4−, and combinations did not result in improved diagnostic accuracy compared with using individual biomarkers. In terms of diagnostic accuracy, the different performance of CSF Ng on MCI ε4− and MCI ε4+ may be due to the fact that less MCI ε4− subjects would progress to AD dementia, while more MCI ε4+ subjects would progress to AD dementia. It is well known that tau protein is mainly distributed in the soma and axons of neurons (Mandelkow, 1999; Wang and Mandelkow, 2016; Mondragón-Rodríguez et al., 2020). However, recent evidence suggested that tau is also a dendritic protein (Mondragón-Rodríguez et al., 2012a,b, 2020; Regan and Piers, 2015). Specifically, under physiological conditions, endogenous tau is located at the postsynaptic of neurons (Mondragón-Rodríguez et al., 2012b, 2020). This raises an important question: What is the role of tau protein in postsynapses (Mondragón-Rodríguez et al., 2020)? According to recent data, dendritic tau seems to regulate the plastic mechanisms related to memory storage (Mondragón-Rodríguez et al., 2012b, 2020; Regan and Piers, 2015; Ittner et al., 2016). At the physiological and molecular levels, lasting changes in synaptic plasticity are considered to be cell-related factors for memory storage. In the phenomenon of synaptic plasticity related to memory, synaptic strength can be long-term-potentiation (LTP) or long-term depression (LTD), and these changes can last for hours to days (Mulkey et al., 1993; Collingridge et al., 2010; Lüscher and Malenka, 2012; Connor and Wang, 2016). The cellular mechanism of LTD is triggered by the activation of synaptic N-methyl-D-aspartate receptors (Mulkey et al., 1993; Collingridge et al., 2010; Lüscher and Malenka, 2012; Connor and Wang, 2016; Mondragón-Rodríguez et al., 2020). Ng is a potential synaptic biomarker. Ng is a post-synaptic protein that is predominantly expressed in the cortex and hippocampus, where it is located in dendritic spines, as well as plays a critical role in regulating LTP and learning (Wellington et al., 2018). Therefore, the diagnostic accuracy was similar between CSF Ng and tau individually and combined together because they may capture different elements of the same neurodegenerative processes in AD. In addition, CSF Ng did not significantly predict conversion from CN to MCI or AD and from MCI to AD, indicating that CSF Ng may be not sensitive in predicting progression in cognitively normal subjects or MCI patients.

Neurogranin is a post synaptic protein involved in memory consolidation as well as a potential biomarker of cognitive decline and neurodegeneration in AD (Huang et al., 2004; Mavroudis et al., 2019). However, the correlation between CSF Ng and cognitive evaluation scores as measured by MMSE and ADAS-cog is inconsistent. De Vos et al. (2015) reported no correlations between CSF Ng and clinical parameters, including MMSE scores (at baseline), nor the annual change in MMSE or disease duration in control, MCI, and AD groups. Hellwig et al. (2015) also reported that the MMSE scores did not associate with Ng concentrations in every group. Portelius et al. (2015) found that CSF Ng did not relate to baseline MMSE and ADAS-cog scores, but high CSF Ng concentrations in MCI individuals who later developed dementia related significantly to a more rapid elevation in ADAS-cog scores over time. Mattsson et al. (2016) demonstrated that Ng was correlated with worsening MMSE and ADAS-cog scores during follow-up in Aβ positive subjects. In the present study, we only collected baseline scores of MMSE and ADAS-cog 11 due to large amounts of missing data due to follow-up. We found that CSF Ng negatively associated with MMSE scores at baseline in the MCI ε4+ group, whereas CSF Ng did not significantly relate to baseline scores of ADAS-cog 11 in any diagnostic group. Although the association between Ng and amyloidosis still needs further validation, there have been some reports indicating a significant effect of amyloidosis on Ng. Therefore, in order to avoid the influence of Aβ42, Aβ42 was taken as a covariate in the above analysis. This result again suggests that the pathophysiological effects of CSF Ng in MCI may be related to specific effects of *APOE* ε4 rather than amyloidosis. Finally, we tested whether CSF Ng related to hippocampal and ventricular volumes as measured by magnetic resonance imaging (MRI) and to cortical glucose metabolism as measured with FDG-PET. CSF Ng did not relate to baseline FDG-PET or ventricular volumes in different diagnostic groups, but CSF Ng was associated with hippocampal volumes in the MCI ε4−, MCI ε4+, and AD ε4+ groups. In this respect, the role of CSF Ng in MCI is not found to be related to *APOE* ε4, possibly through amyloid-independent mechanisms.

The present study has several limitations. Firstly, this study did not investigate non-AD neurodegenerative diseases. Secondly, the ADNI database consists of highly educated individuals who are motivated to participate in research focused on AD. Finally, this study lacks follow-up data and correspondingly cannot examine longitudinal relationships between CSF Ng, *APOE* ε4, neurodegeneration and cognition.

## Conclusion

In conclusion, CSF Ng concentrations were significantly increased in *APOE* ε4 carriers compared to *APOE* ε4 non-carriers with MCI. In addition, CSF Ng identified MCI ε4+ versus CN ε4−, but not MCI ε4− versus CN ε4−. Similarly, CSF Ng negatively related to MMSE scores at baseline in MCI ε4+ subjects but not in MCI ε4− subjects. However, we did not observe the similar phenomena between CN ε4− and CN ε4+, or between AD ε4− and AD ε4+. We propose that the roles of CSF Ng in the pathophysiology of MCI may be related to *APOE* ε4. Future studies will further explore the relationship between *APOE* ε4 and CSF Ng and related mechanisms, providing more evidence for the potential roles of Ng in clinical research, trials and practice of AD and other neurodegenerative diseases.

## Members of the Alzheimer’s Disease Neuroimaging Initiative

Data collection and sharing for this project was funded by the Alzheimer’s Disease Neuroimaging Initiative (ADNI) (National Institutes of Health Grant U01 AG024904) and DOD ADNI (Department of Defense award number W81XWH-12-2-0012). ADNI is funded by the National Institute on Aging, National Institute of Biomedical Imaging and Bioengineering, and through generous contributions from the following: AbbVie, Alzheimer’s Association, Alzheimer’s Drug Discovery Foundation, Araclon Biotech, BioClinica, Inc., Biogen, Bristol-Myers Squibb Company, CereSpir, Inc., Cogstate; Eisai Inc., Elan Pharmaceuticals, Inc., Eli Lilly and Company, EuroImmun, F. Hoffmann-La Roche Ltd., and its affiliated company Genentech, Inc., Fujirebio, GE Healthcare, IXICO Ltd., Janssen Alzheimer Immunotherapy Research and Development, LLC., Johnson & Johnson Pharmaceutical Research and Development, LLC., Lumosity, Lundbeck; Merck & Co., Inc., Meso Scale Diagnostics, LLC., NeuroRx Research, Neurotrack Technologies, Novartis Pharmaceuticals Corporation, Pfizer Inc., Piramal Imaging, Servier, Takeda Pharmaceutical Company, and Transition Therapeutics. The Canadian Institutes of Health Research is providing funds to support ADNI clinical sites in Canada. Private sector contributions are facilitated by the Foundation for the National Institutes of Health (www.fnih.org). The grantee organization is the Northern California Institute for Research and Education, and the study is coordinated by the Alzheimer’s Therapeutic Research Institute at the University of Southern California. ADNI data are disseminated by the Laboratory for Neuroimaging at the University of Southern California.

## Data Availability Statement

The datasets presented in this study can be found in online repositories. The names of the repository/repositories and accession number(s) can be found below: Data used in preparation of this article were obtained from the ADNI database, http://www.adni-info.org.

## Ethics Statement

Written informed consent was obtained from the individual(s) for the publication of any potentially identifiable images or data included in this article.

## Author Contributions

YF: study concept, design, analysis, and interpretation of data, composition of figures, and manuscript drafting. YG: study design, composition of figures, manuscript drafting, and critical review of manuscript for intellectual content. JT: collection of data and manuscript draft. JL and MB: analysis and interpretation of data. HZ: study concept, design, study supervision, and critical review of manuscript for intellectual content. All authors read and approved the final manuscript.

## Conflict of Interest

The authors declare that the research was conducted in the absence of any commercial or financial relationships that could be construed as a potential conflict of interest.

## Footnotes

1 adni.loni.usc.edu

2 www.adni-info.org

## Abbreviations

A β: amyloid- β
AD: Alzheimer’s disease
ADAS-cog: Alzheimer’s disease assessment scale-cog
ADNI: Alzheimer’s disease Neuroimaging Initiative
ANOVA: Analysis of covariance
*APOE*: apolipoprotein E
AUC: area under the curve
CDR: Clinical Dementia Rating scale
CN: cognitively normal
CSF: cerebrospinal fluid
FDG-PET: 18F-fluorodeoxyglucose-PET
HR: hazard ratios
MCI: mild cognitive impairment
MMSE: Mini-mental State Examination
MRI: magnetic resonance imaging
NFT: neurofibrillary tangles
Ng: neurogranin
PET: positron emission tomography
ROC: receiver operating curve
SUVR: standardized uptake value ratio.
